# Engineering the polyphenolic biosynthetic pathway stimulates metabolic and molecular changes during fruit ripening in “Bronze” tomato

**DOI:** 10.1093/hr/uhac097

**Published:** 2022-04-22

**Authors:** Aurelia Scarano, Carmela Gerardi, Eduardo Sommella, Pietro Campiglia, Marcello Chieppa, Eugenio Butelli, Angelo Santino

**Affiliations:** ISPA-CNR, Institute of Science of Food Production, C.N.R. Unit of Lecce, 73100 Lecce, Italy; ISPA-CNR, Institute of Science of Food Production, C.N.R. Unit of Lecce, 73100 Lecce, Italy; Department of Pharmacy, School of Pharmacy, University of Salerno, 84084 Fisciano (SA), Italy; Department of Pharmacy, School of Pharmacy, University of Salerno, 84084 Fisciano (SA), Italy; Department of Public Health, Experimental and Forensic Medicine, Dietetics and Clinical Nutrition Laboratory, University of Pavia, Pavia, Italy; John Innes Centre, Colney Research Park, NRA4 7UH Norwich, UK; ISPA-CNR, Institute of Science of Food Production, C.N.R. Unit of Lecce, 73100 Lecce, Italy

## Abstract

The metabolic engineered Bronze tomato line is characterized by the constitutive over-expression of the *VvStSy* gene encoding a structural protein responsible for the stilbenoids biosynthesis and the fruit-specific over-expression of *AmDel*/*Rosea1* and *AtMYB12* genes encoding transcription factors that activate the polyphenol biosynthetic pathway. This tomato line is known for the increased levels of polyphenols in ripe fruits and for beneficial health promoting antioxidant and anti-inflammatory effects. In this study we analyzed the transcriptional and metabolic profiling in mature green, breaker, orange and ripe fruits compared to the normal tomato counterparts during ripening, to unravel the effect of regulatory and structural transgenes on metabolic fluxes of primary and secondary metabolisms. Our results showed that the *shikimate synthase* (*SK)* gene was up-regulated in the Bronze fruit, and the transcriptional activation is consistent with the metabolic changes observed throughout fruit ripening. These results paralleled with a reduced level of simple sugars and malate, highlighting the consumption of primary metabolites to favor secondary metabolites production and accumulation. Finally, carotenoids quantification revealed a change in the lycopene/β-carotene ratio in the Bronze fruit as a consequence of significant lower level of the first and higher levels of the latter. The high polyphenols and β-carotene content displayed by the Bronze fruit at the later stages of fruit ripening renders this line an interesting model to study the additive or synergic effects of these phyto-chemicals in the prevention of human pathologies.

## Introduction

In recent years, a classic metabolic engineering approach intended as a “direct improvement of product formation through the modification of specific biochemical reactions or the introduction of new ones” [6, 24] has been used successfully in many plant species [16, 28]. However, crops and plants with increased nutritional value have been tested rigorously in animal models only rarely [28], as for the anthocyanin-enriched “purple tomato” [1] or the Bronze tomato producing high levels of flavonols, anthocyanins and stilbenes in the whole fruit [21] as a result of the over-expression of three regulatory genes encoding transcription factors (*AtMYB12, AmDel and AmRos1*) and a structural gene, *stilbene synthase* (*StSy*) (Suppl. Fig. 1).

The transcription factor (TF) AtMYB12 was originally identified as transcriptional activator of flavonol biosynthesis in *Arabidopsis thaliana* [18]. In tomato fruit, *AtMYB12* activates the caffeoylquinic acid and flavonol biosynthetic pathways [14]. Delila (Del) TF encodes a basic helix–loop–helix transcription factor, whereas Rosea1 (Ros1) encodes a MYB-related transcription factor that interact to induce anthocyanin biosynthesis in snapdragon flowers. Their over-expression in tomato under the control of the fruit-specific promoter E8 was reported to be sufficient to stimulate anthocyanin biosynthesis, leading to purple-coloured whole fruit [1].

In addition to these three TFs, the Bronze line also contains a structural gene encoding *stilbene synthase* (*StSy*) from grape [8]. The constitutive over-expression of this gene was able to introduce a new branch in the phenylpropanoid biosynthetic pathway allowing the production of stilbenes (e.g. resveratrol and its derivatives), which are normally absent in tomato fruit.

As a result of this multilevel metabolic engineering approach, mature fruits from the Bronze line were highly enriched in flavonols (about 14.5 mg/g dried weight, about 30 times in comparison with WT fruit), anthocyanins (about 2.5 mg/g dried weight) and stilbenes (about 5.5 mg/g dried weight), whereas no significant changes were observed in the total carotenoids content [21]. These data coincided with a significantly higher anti-oxidant capability (about 7-fold higher than in ripe WT fruit) and a remarkable anti-inflammatory capability, as assessed *in vitro* (bone marrow dendritic cells challenged with LPS) and *in vivo* using mice models of inflammatory bowel disease [3, 12, 21, 22].

Besides the substantial changes in the concentration of different classes of polyphenols, the metabolic engineering approach used to develop the Bronze line may affect, directly or indirectly, other biosynthetic pathways and physiological processes during fruit ripening. For instance, [26] showed that AtMYB12 was able to induce the re-direction of carbon supply from primary metabolism to shikimate and phenylalanine pathways, thus providing more aromatic amino acids for secondary metabolism.

In the present paper, we investigate the transcriptional and metabolic remodeling occurring in Bronze tomatoes during fruit ripening. Together with the predicted changes associated with the phenylpropanoid pathways, we also analyzed the content of sugars and organic acids associated with fruit quality and the effects on carotenoid metabolism.

## Methods

### Plant materials

*Solanum lycopersicum* Mill. cv. Money maker as the control wild type (WT), and the near-isogenic tomato Bronze line, obtained as previously described [21], were used in this study. The plants were grown in greenhouse at 23–25°C under 16 h light and 8 h dark cycle, and managed routinely. The tomato ripening stages were monitored in both WT and transgenic lines, and divided according to the fruit color and days post-anthesis (DPA). MG (Mature green) fruits were defined as green and shiny fully expanded fruits and with no obvious color change, at 35 DPA in WT and 40 DPA in Bronze. B (Breaker) fruits were defined as yellow-colored fruits in WT and pale brown fruits in Bronze, at 50 DPA and 53 DPA, respectively. B + 7 (breaker +7 days) was defined as orange fruit in WT and brown colored fruit in Bronze, at 57 DPA and 60 DPA, respectively. R (Ripe) fruits were characterized by a red color in WT and dark brown in Bronze, and defined at 75 DPA and 80 DPA, respectively. All plant samples were cut, immediately frozen in liquid nitrogen and stored.

### Sample preparation, extraction, detection and quantification of polyphenols

Frozen samples were grinded and extracted two times in 5 ml methanol: water 80:20 (v/v). The extracts were used for the RP-HPLC analyses, for the determination of the content of polyphenols. Flavonoids were detected at 320 nm and stilbenes at 306 nm, as described [21]. For anthocyanins determination, the methanol extracts were appropriately diluted and the pH differential method was used, as described in [10].

### Total polyphenols content

Total content of polyphenols was spectrophotometrically determined according the Folin–Ciocalteu method [15, 23].

### Antioxidant activity

TEAC assay was performed as previously reported [22]. The absorbance of Trolox standard or extracts mixed with diluted ABTS was assayed at 734 nm.

### Brix degrees, sugars and organic acids

Sugars and organic acids were extracted twice by mixing 2.5 g of fresh powder in 5 mL of Milli-Q-water for 1 hour at room temperature. After centrifugation (10 min at 4000 × g), the supernatants were combined and further centrifuged (10 min at 15000 × g). The Brix degrees were determined using a DBR95 Digital Refractometer and the measurements were recorded from two replicates of three independent experiments. Sugar and organic acids were quantified by the HPLC analysis, following the method reported in Gerardi et al. [7].

### Carotenoid content

Dry samples were used for carotenoid quantification using the method described in [20]. Lycopene, lutein and β-carotene were used as external standards for carotenoids quantification.

### RNA extraction and quantitative real-time PCR analysis

Total RNA was isolated from frozen tissues using SV Total RNA Isolation System following the manufacturer’s instruction (Promega Corporation). 1 μg of total RNA was used to synthesize cDNA with SensiFast cDNA Synthesis kit (Labgene Scientific). RT-qPCR analysis of cDNA was performed on a StepOne™ Real-Time PCR System (Applied Biosystem), according to the method described in Lia et al. [11]. The PCR efficiency of each oligonucleotide pair was calculated from linear regressions of the standard curves. The relative quantification of gene expression was established using the comparative 2^−ΔΔCT^ method [19] and ubiquitin gene (UBI) as internal control. Relative gene expressions were normalized to the sample with the lowest relative expression (mature green wild type). Student’s t-test (^*^p < 0.05, ^**^p < 0.01, ^***^p < 0.001) was used to determine the significant difference of relative expression of individual genes among Bronze and wild type tomato fruits. The results are based on at least three replicates in three independent experiments.

### Statistical analysis

Values were expressed as mean ± SEM of three independent experiments. Unless specifically described, two-tailed Student’s t-tests were used.

## Results

### Quality-related features in Bronze tomato undergo changes throughout fruit development

**Figure 1:**
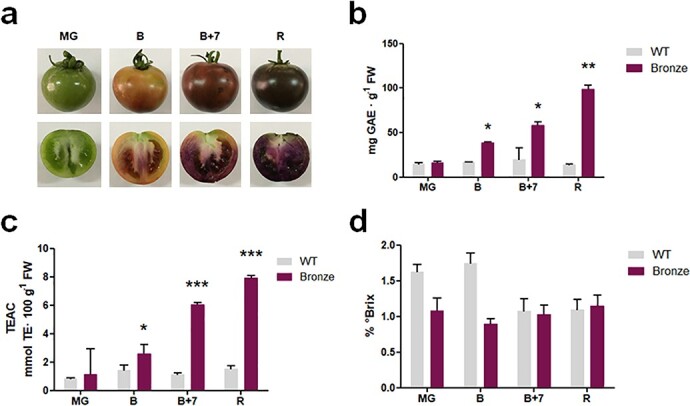
a) Bronze tomato ripening stages (MG, mature green; B, breaker; B + 7, breaker +7 days; R, ripe); b) Antioxidant capacity of methanolic extracts from fruits measured by TEAC assay; c) Total content of polyphenols measured by Folin–Ciocalteu; d) Soluble solutes, defined by the Brix degrees, measured in aqueous extracts from fruits at the four ripening stages. Significance assumed at ^*^p < 0.05, ^**^p < 0.01, ^***^p < 0.001.

The Bronze tomato line was developed following a breeding programme previously described [21, 26] to over-express the regulatory genes *AtMYB12, AmDel, AmRos1,* all under the E8 promoter, and the structural gene *VvStSy* under the 35S promoter (Suppl. Fig. 1). To evaluate the nutritional and quality-related properties of Bronze fruit, we initially measured the total content of polyphenols and antioxidant capacity of methanolic extracts from fruit harvested at four different ripening stages (Fig. 1 a). As showed in Figure 1b, Bronze fruit showed a higher content of total polyphenols in comparison to the near-isogenic Money Maker control fruit collected at the same ripening stages. The content was significantly higher than in WT fruit (p < 0.05) already at the breaker stage, when the ripening process starts and the E8 promoter is active. A similar trend was observed for the anti-oxidant profile measured using TEAC assay. Again, starting from breaker stage, Bronze fruit showed a significantly higher capability (Fig. 1c).

**Figure 2:**
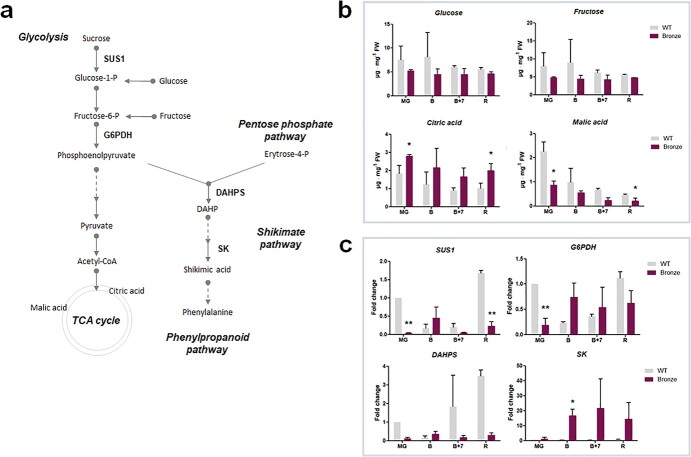
a) Schematic representation of the metabolites and main enzymatic steps genes of the primary metabolism analyzed in this study. Broken arrows indicate several consecutive steps; b) Glucose, fructose, citric and malic acid content quantified by the HPLC methods; c) Gene expression profiling measured by Real time qPCR in samples from mature green (MG), breaker (B), breaker +7 days (B + 7) and ripe (R) fruits in WT and Bronze tomato line (n = 3). Biosynthetic genes are named according to the following abbreviations: *SUS1*, sucrose synthase 1; *G6PDH,* glucose-6-phosphate dehydrogenase; *DAHPS*, 3-deoxy-d-arabino-heptulosonate 7-phosphate synthase; *SK,* shikimate synthase.

To estimate the soluble solutes, including the levels of acids and sugars, we measured the Brix index in extracts of fruits collected at different stages of maturation. The Brix degrees recorded in the mature green and breaker stages of the Bronze tomatoes were slightly reduced compared to the WT (Figure 1d), a result which was partially in accordance with the levels of simple sugars and organic acids detected by HPLC method (Figure 2).

### Energy metabolism in Bronze tomato fruit

To evaluate possible effects of the transgenes on energy metabolism, we measured the level of the monosaccharides glucose and fructose and two organic acids, malic and citric, deriving from the tricarboxylic cycle. Glucose and fructose are converted to phosphoenolpyruvate during glycolysis, or, being the latter a precursor of the tricarboxylic cycle, it can also enter the shikimate pathway to produce phenylalanine, the precursor of all the downstream metabolites in the phenylpropanoid pathway (Figure 2a).

The levels of glucose and fructose in Bronze fruit were fairly stable throughout the whole ripening process and were always lower compared to WT, particularly at early stages (MG and B stages). Malic acid was also reduced along the ripening stages with levels significantly lower at the MG and R stages when compared to WT. Conversely, the level of citric acid was significantly higher in the Bronze tomato, at the same ripening stages (Figure 2b).

To obtain more clues on these metabolic changes, we carried out an expression profiling of two genes related to the energy metabolism, namely *SUS1* (*Sucrose synthase 1*) and *G6PDH* (*glucose-6-phosphate dehydrogenase*). Two other genes, *DAHPS* (*3-deoxy-d-arabino-heptulosonate 7-phosphate synthase*) and *SK* (*shikimate synthase*) were chosen for their involvement in the shikimate pathway (Fig. 2a). Our analysis indicated that *SUS1* was significantly down-regulated in Bronze fruit at MG and R stages. Similarly to *SUS1*, also *G6PDH* was repressed at the MG stage (Fig. 2c).

A contrasting behavior was observed for the two genes involved in the shikimate pathway: *DAHPS* was highly repressed while *SK* was up-regulated in Bronze fruit compared to WT fruit at the same ripening stages.

### Polyphenol content, flavonoid metabolism and transporter-related genes expression profiling

**Figure 3:**
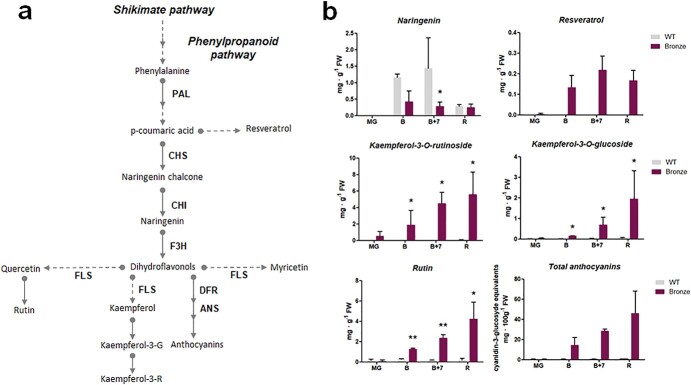
a) Schematic representation of the phenylpropanoid and flavonoid biosynthetic pathway; b) Polyphenol content measured by HPLC in extracts from mature green (MG), breaker (B), breaker +7 days (B + 7) and ripe (R) fruits in WT and Bronze tomato line (n = 3). Significance assumed at ^*^p < 0.05, ^**^p < 0.01.

The metabolic profiles of the main classes of polyphenols in Bronze fruit during ripening are reported in Figure 3. In agreement with the measurement of the total polyphenol content (Figure 1c), quantitative HPLC analysis indicated a significant increase in the levels of kaempferol-3-rutinoside, kaempferol-3-glucoside and rutin in Bronze fruit compared to WT. The content of naringenin, the most abundant flavonoid in WT tomato, was lower at all the ripening stages in Bronze fruit (with a content significantly lower than WT at B + 7 stage). Stilbenes and anthocyanins were only detected in Bronze fruit, where they increased during ripening.

**Figure 4:**
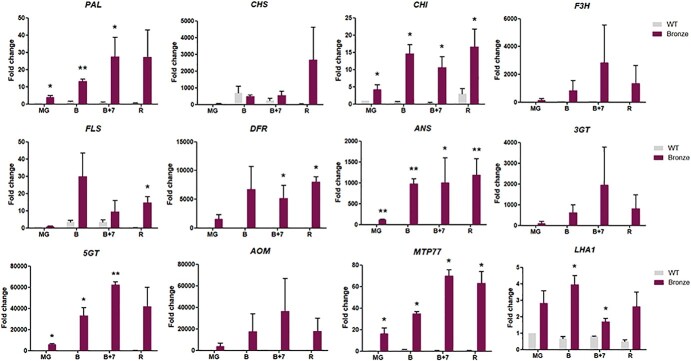
Expression profiles of genes involved in the polyphenol metabolism. Gene expression was measured by Realtime qPCR in samples from mature green (MG), breaker (B), breaker +7 days (B + 7) and ripe (R) fruits in WT and Bronze tomato line (n = 3). *PAL*, phenylalanine ammonia lyase; *CHS*, chalcone synthase; *CHI*, chalcone isomerase; *F3H*, flavanone-3-hydroxylase; *FLS*, flavonol synthase; *DFR*, dihydroflavonol reductase; *ANS*, anthocyanidin synthase; *3GT*, flavonol-3-glucosyltransferase; *5GT*, anthocyanin-5-glucosyltransferase; *AOM*, anthocyanin O-methyl-transferase; *MTP77* (anthocyanin permease); *LHA1* (plasma membrane H^+^-ATPase). Significance assumed at ^*^p < 0.05, ^**^p < 0.01.

To further characterize the molecular changes occurring in Bronze fruit, the expression profile of the main structural genes along the flavonoid pathway (*PAL, CHS1, CHI, F3H, FLS, DFR, ANS*) was monitored. We found that most of the structural genes encoding the enzymes driving the biosynthetic flux towards flavonols and anthocyanins were significantly up-regulated, mainly from the B stage, indicating that both the upstream and downstream part of the biosynthetic pathway are activated. We also verified the expression of some genes encoding enzymes involved in the decoration of anthocyanins (catalyzing for example glycosylation and methylation), such as *3GT*, *5GT* and *AOM.* The expression of *3GT*, *5GT* and *AOM* was undetectable or showed very low levels in WT fruit while, in the Bronze line, all these genes showed a similar trend peaking at the B + 7 ripening stage, where higher transcript levels were recorded (Fig. 4).

We finally investigated the expression of two genes encoding transporters involved in vacuolar anthocyanin sequestration (MTP77) or proton translocation (LHA1). MTP77 has been proposed as a putative vacuolar anthocyanin transporter (PAT) functioning as an antiporter pumping protons outside the vacuole [2, 17]. LHA1 is a tomato plasma membrane H^+^-ATPase, which has been hypothesized to be involved in protons extrusion outside the cell [5]. Our results clearly showed a significant increase in the expression of both genes. In the case of *MTP77* a significant increase was observed at later ripening stages (B + 7 and R), whereas *LHA1* expression peaked at the B stage and remained sustained to the R stage. (Fig. 4).

### Carotenoid content and carotenoid-related gene expression profiling

**Figure 5:**
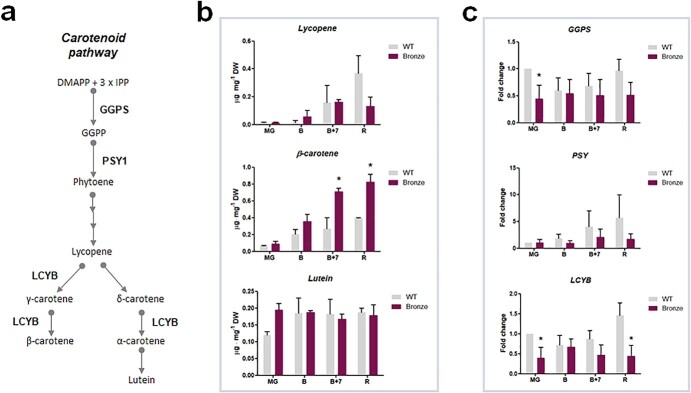
a) Schematic representation of the carotenoid biosynthetic pathway; b) Carotenoid content quantified by HPLC methods; c) Expression profiles of biosynthetic genes measured by Real time qPCR in samples from mature green (MG), breaker (B), breaker +7 days (B + 7) and ripe (R) fruits in WT and Bronze tomato line (n = 3). Biosynthetic genes are named according to the following abbreviations: *GGPS*, geranyl-geranyl pyrophosphate synthase; *PSY1*, phytoene synthase 1; *LCYB*, β-lycopene cyclase 1. Significance assumed at ^*^p < 0.05.

We evaluated the content of the main carotenoids (lycopene, β-carotene and lutein) and the transcript levels of some of the key genes involved in the carotenoid pathway in tomato. As shown in Figure 5, Bronze fruit contained a significantly higher level of β-carotene compared to WT at the late stages of ripening. Similar results were recorded on the ripe fruits of the parental lines MYB12, MYB12/Del/Ros and MYB12/StSy (Suppl. Fig. 3). This increase was achieved at the expenses of lycopene, thus resulting in a sharp change in the lycopene/β-carotene ratio compared to WT tomato (Suppl. Fig. 2). Lycopene content was similar to that of WT fruit during the early stages up to the B + 7 stage, but was reduced in ripe fruit. Conversely, the β-carotene content was significantly higher in the B + 7 and R stages in Bronze tomato. Finally, similar levels were recorded for lutein in fruit of the two lines. The transcriptional profiling revealed that the expression of *GGPS* and *PSY*, two genes involved in the upstream part of carotenoid biosynthesis pathway, was down-regulated in Bronze (mainly at the MG and R stages), with a significant decrease recorded for *GGPS* expression at the MG stage. Finally, *LCYB*, a gene encoding an enzyme involved in the lycopene cyclization, was also down-regulated, with a significant reduction in Bronze tomato fruit at the MG and R stages.

## Discussion

### The synthesis of polyphenols in the bronze fruit is sustained by carbon and energy metabolisms

In this study, we analyzed the metabolic fluxes occurring during the ripening process of Bronze tomato fruit compared to the WT counterparts. Results here presented pointed to a gradual increase in polyphenols and antioxidant capacity from the breaker stage and reaching the highest levels at the ripe stage.

Together with the onset of polyphenol biosynthesis and accumulation, a reduction in the levels of glucose, fructose and malic acid was observed, mainly at early ripening stages. The reduction in simple sugars observed in the Bronze fruit confirms that carbon and energy supply deriving from primary metabolism represents an important sink employed to fuel the shikimate and phenylpropanoid pathways, as also suggested by the upregulation of the early genes of the pathway, i.e. *SK* and *PAL*. The observed reduction in naringenin, a metabolic precursor of flavonoids, together with the increase in citric acid and a drastic consumption of malic acid (Figures 2 and 3) could indicate a continuous supply of ready-to-use precursors and energy along the entire ripening process and confirm the key role of MYB12 TF in the activation of the phenylpropanoid pathway [26].

Interestingly, we have noticed that genes related to the energy metabolism, such as *SUS1, G6PDH* and *SK*, are over-expressed in the Bronze fruit at the breaker stage, whereas most of the genes implicated in the flavonoid biosynthetic pathway (*PAL, CHS, CHI, F3H, FLS, DFR, ANS*) and flavonoid/anthocyanin decoration (*3GT, 5GT, AOM*) peaked at a later stage (B + 7 stage). Therefore, the expression of genes involved in primary or secondary metabolism could be subjected to a different temporal regulation, with an initial activation of energy-related pathways followed by the activation of secondary metabolism. Such mechanism could also reflect the ethylene-dependent regulatory pattern for the phenylpropanoid production during fruit ripening, as also witnessed by the presence of ethylene responsive elements in the promoter region of E8.

Another interesting aspect of our study is the up-regulation of the MATE (Multidrug and Toxic compound Extrusion, or Multi-Antimicrobial Extrusion) MTP77, previously reported as a putative anthocyanin permease [17] working as a H^+^-antiporter system.

The dramatic upregulation of this gene, which matches the expression pattern of other genes specifically involved in anthocyanin biosynthesis, supports the role of MTP77 as an anthocyanin vacuolar transporter.

On the other hand, the expression of a different gene, Solyc12g006360, previously proposed to encode a putative anthocyanin MATE transporter [4], was below the detection limit in our analysis (data not shown), suggesting that this gene is unlikely to be involved in the transport of anthocyanins into the vacuole.

Our analyses also showed an up-regulation of *LHA1* during Bronze fruit ripening. This gene encodes a P-type H^+^-ATPase localized on the plasma membrane and is involved in protons translocation outside the cytosolic compartment. A possible explanation for its upregulation might be related to the large release of protons into the cytosol resulting from the MTP77-mediated sequestration of anthocyanins into the vacuole. In this context, LHA1 might be required for cytoplasmic pH homeostasis by pumping protons outside the cell.

### The lycopene/β-carotene ratio is altered in bronze fruit

Quantitative analyses of carotenoids indicated that the observed reduction in lycopene content in Bronze fruit compared to WT was largely balanced by a significative increase in β-carotene (at the B + 7 and R stages), making the total carotenoid content in this line similar to wild-type but with a change in the lycopene/β-carotene ratio (Suppl. Fig. 2). A similar trend was observed in the parental lines MYB12, MYB12/Del/Ros and MYB12/StSy, thus indicating that MYB12 TF can have an impact in the lycopene/β-carotene ratio (Suppl. Fig. 3).

All the three structural genes involved in carotenoid biosynthesis considered in this study (*GGPS*, *PSY* and *LCYB*) were downregulated in Bronze fruit compared to WT, although this was significant only for *LCYB*. Therefore, the low content of lycopene in Bronze fruit might be the result of the coordinated reduced expression of several carotenoid biosynthetic genes. On the other hand, the increase in β-carotene could be associated with a low rate of catabolism for this phytochemical, essential for photosynthetic tissues [13]. The degradation of carotenoids can proceed by enzymatic or non-enzymatic routes, and it is known that the latter process is initiated by reactive oxygen species (ROS) [13]. We hypothesize that the increased content of polyphenols in Bronze tomatoes and the parental lines MYB12/Del/Ros and MYB12/StSy, as also witnessed by the increased anti-oxidant capability (Suppl. Fig. 3), can act as ROS scavenger and affect several physiological processes during fruit ripening.

In this context, the observed increase in β-carotene could be the result of delayed ripening [27] and reduced degradation of carotenoids, both mediated by ROS.

### The Bronze tomato is a good model to study the combination of polyphenols and β-carotene in inflammation-based human diseases

The high level of β-carotene, together with the increased content in flavonols, anthocyanins and stilbenes, offer the opportunity to use Bronze tomatoes as an interesting model to study the combined effect of polyphenols and pro-vitamin A for the prevention human pathologies sharing a common inflammation base. It is noteworthy that, according to our quantitative analyses, the β-carotene content in two Bronze fruits could be sufficient to provide 100% retinol RDA (about twice the β-carotene content of 100 g golden rice; [25]). This could be achieved even before full ripeness (B + 7), when the content of β-carotene is more than doubled compared to wild type and the content of polyphenols is already very high.

Preliminary results (Santino et al; manuscript in preparation) indicate a strong inhibitory effect exerted by the methanolic extracts of fruit at both ripening stages (B + 7 and R) on the production of some pro-inflammatory interleukins while, in a previous study, we have documented the beneficial effects of Bronze tomatoes in reducing the symptoms of inflammatory bowel disease (IBD), when included at 1% in a standard mouse diet [21]. On the basis of the molecular and metabolic characterization of Bronze fruit in the present study, it is possible that some of the protective properties of the Bronze tomato might be in part mediated by β-carotene. Anti-inflammatory effects have been already reported for this compound at concentrations ranging 25 to 100 μM, with an effect greater than that exerted by lycopene [9].

## Conclusions

The results here reported indicate that fruit ripening in Bronze tomatoes is accompanied by major transcriptomic and metabolic changes impacting on the nutritional quality of ripe fruit, and confirm that this line can be considered as an interesting model for the study of the molecular crosstalk between carotenoid and polyphenols pathways.

From the nutritional point of view, the availability of near isogenic lines, as in the case of the Bronze tomato, accumulating high levels of different classes of biocompounds, offers a great opportunity to study their additive or synergic effects in the prevention of important human pathologies using a single food matrix context, thus providing more reliable results than those obtained with purified compounds.

## Acknowledgments

The authors acknowledge Mr Leone D’Amico for technical assistance. This work was in part funded by and the CNR-DiSBA project NutrAge (project nr. 7022).

## Author Contributions

A.Sc. (Aurelia Scarano), C.G., E.S., E.B. acquired the data. A.Sc., C.G., E.S., E.B., A.Sa. (Angelo Santino) analyzed the results. A.Sc., M.C., P.C., E.B. and A.Sa. wrote the paper. All the authors critically revised the manuscript and approved the final version.

## Data availability

The data underlying this article are available in the article and in its online supplementary material.

## Conflicts of Interest

The authors declare no conflict of interest.

## Supplementary data

Supplementary data is available at *Horticulture Research * online.

## Supplementary Material

suppl_data_uhac097
