# Supplementary material for: Engineering the polyphenolic biosynthetic pathway stimulates metabolic and molecular changes during fruit ripening in “Bronze” tomato
Source: Hortic Res. 2022 Apr 22;9:uhac097. doi: 10.1093/hr/uhac097 (PMC9249581; doi:10.1093/hr/uhac097)

**Supplementary Figure 1. Breeding design of Bronze tomato (from Scarano et al. 2018)**

**
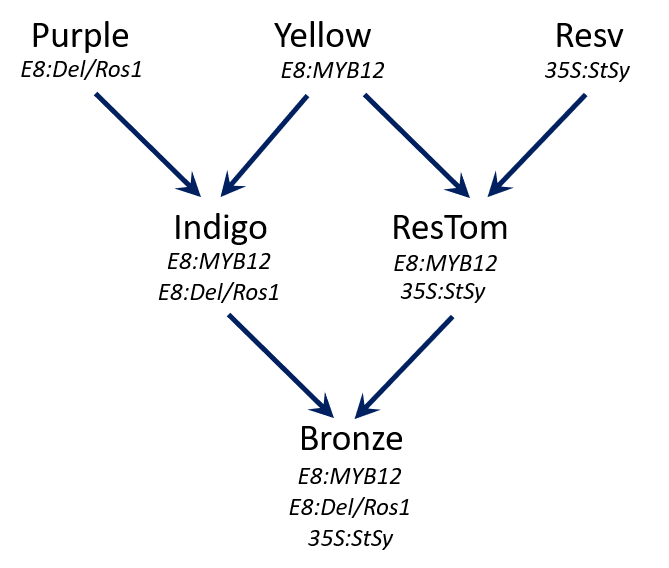
**

**Supplementary Figure 2. Lycopene/β-carotene ratio in Wild type (WT) and Bronze tomato at different ripening stages.**


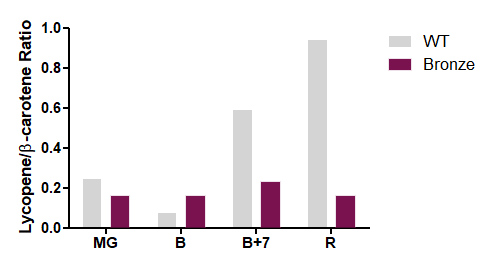


**Supplementary Figure 3. Carotenoids content and antioxidant capacity in the parental lines used to develop the Bronze tomato line**


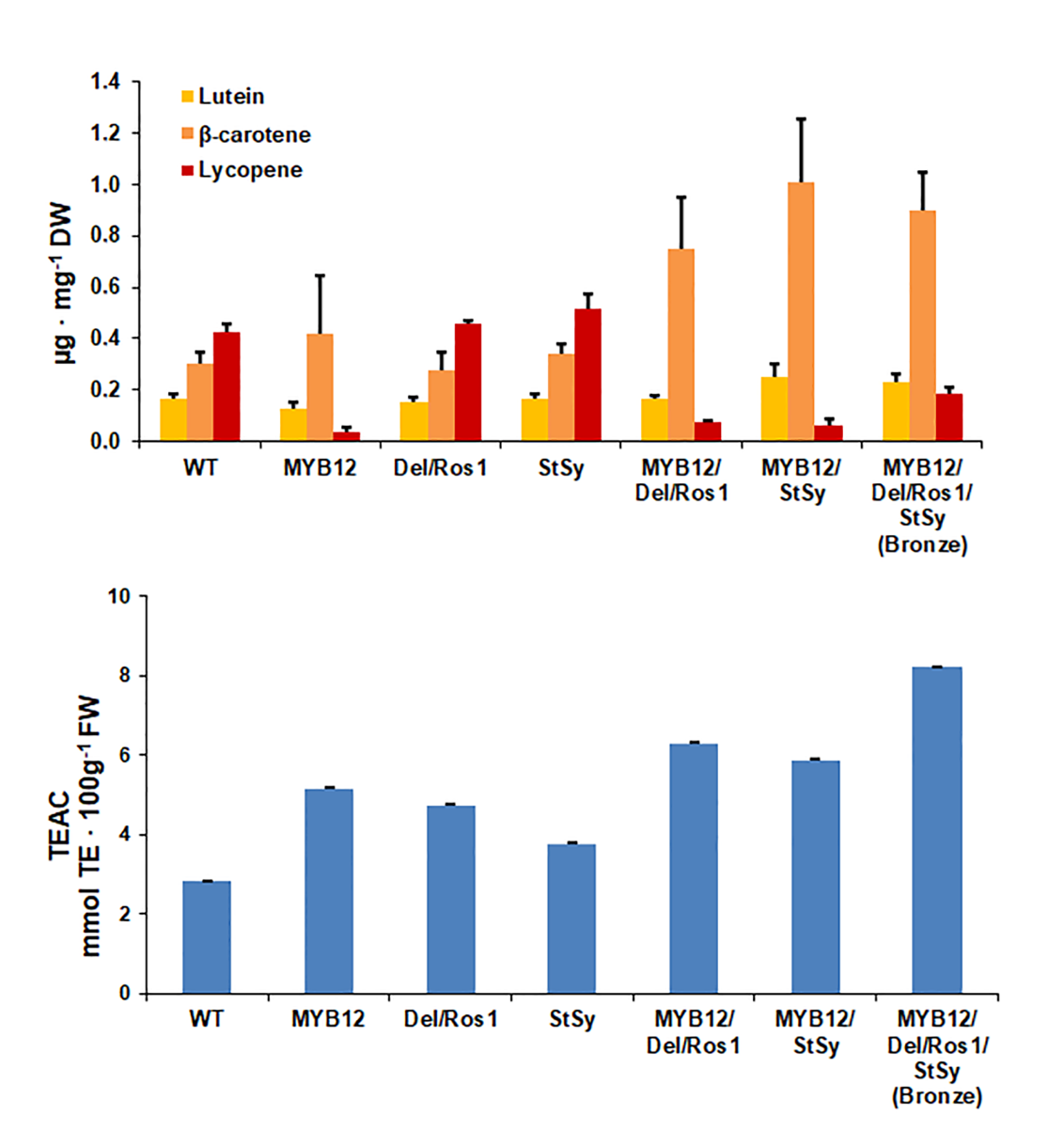

Supplement: suppl_data_uhac097 [file suppl_data_uhac097.zip › Supplementary Figures_Scarano et al..docx]
