# Supplementary material for: Engineering the polyphenolic biosynthetic pathway stimulates metabolic and molecular changes during fruit ripening in “Bronze” tomato
Source: Hortic Res. 2022 Apr 22;9:uhac097. doi: 10.1093/hr/uhac097 (PMC9249581; doi:10.1093/hr/uhac097)
Supplement: suppl_data_uhac097 [file suppl_data_uhac097.zip › Supplementary Table 1.docx]

**Supplementary Table 1. Oligonucleotides used in RealTime-qPCR experiments**

| ID | Symbol | Name | Primer sequence (5’-3’) |
| --- | --- | --- | --- |
| Solyc02g093830 | *G6PD* | Glucose-6-phosphate 1-dehydrogenase | FW: GTCAGTGGCTCTTATGATTCTGG  RV: GTCTTCTGGATGATCCTTCTGTG |
| Solyc07g042550 | *SUS1* | Sucrose synthase | FW: GTTAGGTGTAACACAGTGCACCA  RV: AGCTGTGAACTGAGCTGAGAAGT |
| Solyc04g074480 | *DAHPS* | 3-deoxy-7-phosphoheptulonate synthase | FW: CCTTCACTATGATTGCTCTGCTC  RV: TCACTCACCTTAATACCGAGAGG |
| Solyc04g051860 | *SK* | Shikimate kinase | FW: GGACGATGTGTATACCTTGTTGG  RV: CTCTATCAGCCTGTCACAGTCAA |
| Solyc01g056940 | *UBI* | Ubiquitin | FW: GCCAAAGAAGATCAAGCACA  RV: AAGGAGTGCCCTAATGCTGA |
| Solyc10g086180 | *PAL* | Phenylalanine ammonia-lyase | FW: AATTGCTTCGAGTCGTGGATAG  RV: ACAAGGACTTGTCTCAGCTTCTG |
| Solyc09g091510 | *CHS-1* | Chalcone synthase -1 | FW: CCTTTATTTGAACTCGTCTCAGC  RV: CAGGAACATCCTTGAGTAAGTGG |
| Solyc05g010320 | *CHI* | Chalcone-flavonone isomerase | FW: TTGTCAACTCGGTCTAATGTGTC  RV: TAAAGTGGGACCTTATTGCACAC |
| Solyc02g083860 | *F3H* | Flavanone 3-hydroxylase | FW: ATGGATGAGCCGATTACATTTG  RV: TGGCCTCTTCAGTTTGTATCTTC |
| Solyc11g013110 | *FLS* | Flavonol synthase | FW: TGTCCCATATCACCCTTCTTGTC  RV: TCACCAATGTGGACAATTATAGCA |
| Solyc08g080040 | *ANS* | Anthocyanidin synthase | FW: ACGAACAGGATTTTGCTGCT  RV: TTTGAGCTCAGCAACTGCAT |
| Solyc02g085020 | *DFR* | Dihydroflavonol reductase | FW: GACTTGCCGACAGAAGCAAT  RV: GTGCATTCTCCTTGCCACTT |
| Solyc10g083440 | *3GT* | Flavonoid-3-O-glucosyltransferase | FW: ATAAGAGTGTTGGCGTTTTTGTAAC  RV: CCTTAATACTTTCCCTTTTCCTTCA |
| Solyc12g098590 | *5GT* | Anthocyanin-5-O-glucosyltransferase | FW: TGTTCTGAAAATGGGAGTCCT  RV: GCTGGTTGACTCCAAAGAAGA |
| Solyc09g082660 | *AOM* | Anthocyanin-O-methyltransferase | FW: ATCCAAGAGAGCATGAGCTACTAAA  RV: TAGTTAGAAGAGAGTAGCCGGTGAA |
| Solyc03g025190 | *MTP77* | Anthocyanin permease | FW: TGCAAGAAGCTGTTGCTGAT  RV: TAAAGCTTGCCATCCACCTC |
| Solyc03g113400 | *LHA1* | Plasma membrane H^+^-ATPase | FW: AAAGGCAGCTCACCTTGTTG  RV: GACGGTATTTGCGGTGTTG |
| Solyc09g008920 | *GGPS* | Geranyl-geranyl pyrophosphate synthase | FW: GCTGTTGGTGTCTTATATCGTG  RV: CTTCTCAATGCCATAAACGCTG |
| Solyc03g031860 | *PSY1* | Phytoene synthase 1 | FW: GGAAAGCAAACTAATAATGGACGG  RV: CCACATCATAGACCATCTGTTCC |
| Solyc04g040190 | *BLCY1* | β-lycopene cyclase 1 | FW: GTCCACTTCCAGTATTACCTCAG  RV: TGTCCTTGCCACCATATAACC |
